# Habitat constraints on carotenoid‐based coloration in a small euryhaline teleost

**DOI:** 10.1002/ece3.4003

**Published:** 2018-04-02

**Authors:** Francesco Cavraro, Giulia Gheno, Renzo Ganzerla, Matteo Zucchetta, Piero Franzoi, Stefano Malavasi

**Affiliations:** ^1^ Department of Environmental Sciences, Informatics and Statistics Ca’ Foscari University Venice Venezia Mestre Italy; ^2^ Department of Molecular Sciences and Nanosystems Ca’ Foscari University Venice Venezia Mestre Italy

**Keywords:** carotenoids, coastal lagoons, habitat structure, killifish, life history

## Abstract

Display of bright and striking color patterns is a widespread way of communication in many animal species. Carotenoid‐based coloration accounts for most of the bright yellow, orange, and red displays in invertebrates, fish, amphibians, reptiles, and birds, being widely considered a signal of individual health. This type of coloration is under the influence of several factors, such as sexual selection, predator pressure, pigment availability, and light transmission. Fish offer numerous examples of visual communication by means of color patterns. We used a small cyprinodontid fish, *Aphanius fasciatus* (Valenciennes, 1821), as a model species to assess habitat constraints on the color display in male caudal fin. Populations from natural and open/closed artificial habitats were tested for differences in the pigmentation of caudal fins. The most important factors explaining the intensity of coloration were the habitat type and the chlorophyll concentration in the sediment, followed by water turbidity; yellow fins were observed in natural habitats with low chlorophyll concentration and high water turbidity, while orange fins occurred in artificial habitats with high chlorophyll concentration and low turbidity. Furthermore, *A. fasciatus* in artificial habitats showed a higher somatic and a lower reproductive allotment with respect to natural habitats, according to the existing literature on the species. Furthermore, in closed artificial habitats, where the most intense reddish coloration of caudal fins was observed, a trade‐off between somatic growth and the coloration intensity of a carotenoid‐based sexual ornament has been observed; in these populations, intensity of caudal fin coloration was negatively related to the somatic allotment. Results of this study suggested how both the pigmentation of male's caudal fin and the life history strategies of the species are constrained by habitat characteristics.

## 1 INTRODUCTION

Display of bright and striking color patterns to enhance visual signals is a widespread way of communication in many animal species (Burtt, 1979; Cott, 1940; Hailman, 1979; Rowland, 1979). These signals can be used in interspecific communication, for example, sending a message to possible predators, as in the case of aposematic coloration (Endler, 1988). In other cases, these patterns evolved as intraspecific signals, often leading to a marked sexual dimorphism and to seasonal variations in the intensity of the coloration (Andersson, 2000). The pigments used, mainly carotenoids, melanins, and pterins (McGraw, 2005), can be synthetized by the organisms or ingested through the diet (Hill, 1996).

One of the most studied classes of pigments is carotenoids. Carotenoid‐based coloration accounts for most of the bright yellow, orange, and red displays in invertebrates, fish, amphibians, reptiles, and birds (Grether, Hudon, & Millie, 1999; Hill, 1996). Fish are a good model to study how the visual communication by means of changes in color patterns is shaped by natural selection forces, for example, sexual selection, predation pressure, and light transmission (Deutsch, 1997; Endler, 1980; Endler & Houde, 1995; Evans & Norris, 1995; Fuller, 2002; Marshall, 2000; Pike, Blount, Lindstrom, & Metcalfe, 2010).

Animals can only obtain carotenoids through the diet (Latscha, 1990). It is generally assumed that bright yellow, orange, and red carotenoid‐based colorations are honest signals of the general health of an individual (Grether et al., 1999; Johnson & Fuller, 2014; Kodric‐Brown, 1989; Olson & Owens, 1998), giving information about an individual's vigor, resistance to parasites/pathogens, and ability to find food resources (Kodric‐Brown, 1998). Indeed, this coloration is costly to express, as vertebrates are inefficient carotenoid assimilators (Grether et al., 1999), and carotenoids used as pigments cannot contribute to various physiological processes regarding antioxidant protection, immune function, and reproduction (Brown, Leonard, McGraw, & Clotfelter, 2014; McGraw, 2005; McNeil, Friesen, Gray, Aldredge, & Chapman, 2016; Pike et al., 2010; Svensson, Pelabon, Blount, Surai, & Amundsen, 2006). As only a small fraction of the carotenoids ingested through the diet can be assimilated, the availability of these pigments in nature can strongly influence the intensity of such coloration. In the aquatic environment, only Grether et al. (1999), Grether, Hudon, and Endler (2001) investigated this topic, showing how the carotenoid availability limited the sexual coloration of orange spots in male guppies, *Poecilia reticulata*. In particular, these authors found out that carotenoid availability varied geographically, being correlated with the algal standing crop on the river substrates.

In the aquatic environment, studies concerning the carotenoid‐based coloration in fish focused mainly on fresh‐water species, and only a few have investigated the influence of carotenoid availability on fish pigmentation (Grether, 2000; Grether et al., 1999, 2001). No information was found about fish living in transitional water ecosystems. In this study, we used the cyprinodontid *Aphanius fasciatus* (Valenciennes, 1821) as a model species to test for the effects of habitat constraints on the expression of a carotenoid‐based sexual ornament (the male yellow‐orange caudal fin) in a coastal lagoon. As this kind of pigmentation may represent a cost for the organism, we considered also the effect that two biological traits, somatic and reproductive investments, may have in influencing caudal fin coloration.

**Figure 1:**
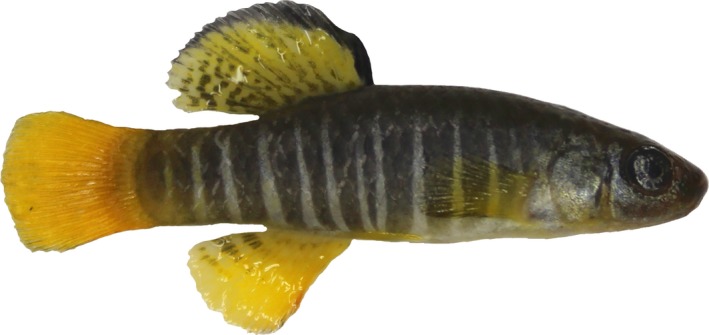
Male specimen of *Aphanius fasciatus* (Valenciennes, 1821)

Males of this species (Figure 1) show bright thin white vertical bars on a dark blue‐gray background and large and modified caudal, anal, and dorsal fins, which display a brilliant coloration (Gandolfi, Zerunian, Torricelli, & Marconato, 1991).

While the dorsal and anal fins show an iridescent yellow pigmentation and are mainly used during intrasexual aggressive displays (Cavraro, Torricelli, & Malavasi, 2013), the caudal fin during the reproductive season is pigmented with a yellow‐orange carotenoid‐based coloration and it is always visible during the courtship. This species shows a circum‐Mediterranean distribution, inhabiting shallow coastal transitional waters, such as estuaries, coastal ponds, and lagoons (Maltagliati, 1999). In the Venice lagoon, it is found in the intertidal creeks crossing salt marshes, but also in artificial small‐sized canals that can be found in some islands of the Venice lagoon (Cavraro, Daouti, Leonardos, Torricelli, & Malavasi, 2014; Cavraro, Torricelli, Franzoi, & Malavasi, 2013). Most of these canals hosted traditional fish‐farming activities but at present are abandoned and partly renaturalized, hosting, in some cases, high fish abundances (Cavraro, Zucchetta, Malavasi, & Franzoi, 2017). The artificial creeks can be divided into two main categories: open systems, directly connected with lagoon waters, and closed systems, isolated from lagoon circulation. The three habitat types (natural, artificial open, and artificial closed creeks) show different structural characteristics and tidal regimes that influence the water transparency, the primary productivity, and the predation pressure (Cavraro, et al., 2014; Cavraro, Torricelli, et al., 2013).

*Aphanius fasciatus* is an omnivorous species, mainly feeding both on benthic diatoms and invertebrates (Leonardos, 2008). Thus, *A. fasciatus* can assume carotenoids directly from benthic microalgae or indirectly through the benthic microinvertebrates. In shallow water habitats, such as the creeks considered in this study, the microphytobenthic community plays a key role among primary producers (MacIntyre, Geider, & Miller, 1996; Webster, Ford, & Hodgson, 2002), may be representing the dominant source of carotenoids for the trophic chain. Nevertheless, to make effective such signal, it must be conveyed through the medium, that is water, in order to reach the receiver. In the aquatic environment, the effectiveness of visual signals can be influenced by the light transmission properties of the water (Endler, 1992; Fuller, 2002; Pauers, 2011). In particular, water turbidity can deeply alter or weaken a visual signal based on a color pattern (Candolin, Salesto, & Evers, 2007; Maan, Seehausen, & Van Alphen, 2010).

Furthermore, *A. fasciatus* life history strategies seem to be related to the structural characteristics of the environment. For example, in closed systems, the female reproductive allotment is lower and partitioned into more reproductive events over the life span than in the open systems (Brigolin, Cavraro, Zanatta, Pastres, & Malavasi, 2016; Cavraro et al., 2014), where fish show a shorter life span, a higher and more peaked reproductive investment, and a lower somatic investment. Thus, life history strategies seem to be related to the structural characteristics of the environment. These characteristics might also influence the expression of a carotenoid‐based sexual ornament, through the physiological and energetic investments related to carotenoid metabolism.

In light of this information, we tested for differences in caudal fin color related to both environmental and biological predictors. We expected that caudal fin pigmentation would vary among habitats influenced by the availability of carotenoids in the environment, quantified through the concentration of sediment chlorophyll, and by water turbidity, in order to maximize the visibility of the signals. At the same time, we expected a negative relationship between the intensity of fin pigmentation and somatic/reproductive investment that would be consistent with a trade‐off in energy allocation.

## 2 MATERIALS AND METHODS

Ten sites (see Appendix S1) were sampled between the end of May and the beginning of June, during the peak of the reproductive period of *A. fasciatus* (Cavraro et al., 2014). Sampling sites were chosen, as in Cavraro et al. (2014), to represent three habitat types: two natural salt marsh creeks and eight artificial creeks divided into two categories: four open subtidal systems and four closed creeks. Natural habitats are intertidal creeks; we expect that the daily water renewal determines a high turbidity and an export of nutrients, thus, reducing the local primary productivity. Furthermore, the complete drainage during low tide phase forces fish to move into deeper water, where they are exposed to piscivorous predators. Artificial open habitats are subtidal creeks that do not drain completely during the low tide phase, thus, providing fish with a shallow water refuge. Furthermore, the reduced tidal influence should determine more transparent water and a minor loss of nutrients. Artificial closed habitats are the most productive habitats (Cavraro, Torricelli, et al., 2013), probably due to the lack of water exchange with the rest of the lagoon that accumulates the nutrients, while the reduced circulation lowers the water turbidity. Furthermore, the isolation prevents from the access of aquatic predators (Cavraro et al., 2014).

The same day of samplings, the water transparency was measured, in two locations in each site, with a turbidity sensor (HI7609829‐4) and microphytobenthos, considered the most important source of carotenoids to the trophic chain (Grether et al., 1999), was sampled by collecting three cores of sediment (28 mm of diameter) in each site. Microphytobenthos standing crop was estimated measuring chlorophyll concentration in the first cm of sediment by fluorimetric determination, using the method proposed by Holm‐Hansen, Lorenzen, Holmes, and Strickland (1965).

Fish were sampled using a small beach seine net, and from 10 to 54 males from each site were sacrificed with an excess of anesthetic (2‐phenoxyethanol), which should have no effect on fish coloration (Kalinowski, Robaina, Fernandez‐Palacios, Schuchardt, & Izquierdo, 2005; McMahon & Hartman, 1988). In each site, all the fish samples were immediately photographed together on a white background in full sunlight, using a reference color chart and a millimetric reference scale that allowed subsequent image analysis. Photographs were analyzed in ImageJ (Schneider, Rasband, & Eliceiri, 2012). First, photographs were split into the three RGB channels in grayscale. For each image, the RGB values of nine colors from the reference chart and of caudal fins of all the sampled males were recorded. Then, the photographs were standardized using the coefficients of a linear regression calculated between the RGB values of the nine reference colors and those in a photo of the color chart used as reference. We assumed that the intensity of caudal fin coloration was linked to the concentration of carotenoids; the shift from yellow to orange coloration would correspond to an increasing accumulation of pigments in the tissue. *A. fasciatus* males usually show a yellow caudal fin (Gandolfi et al., 1991; Malavasi, Georgalas, Cavraro, & Torricelli, 2010), and only some populations exhibit an orange pigmentation. Thus, we choose a redness index to quantify the color shift from yellow to orange‐reddish colorations, according to Levin, Ben‐Dor, and Singer (2005):

$$\text{redness}=\frac{{\text{red}}^{2}}{\text{blue}\cdot {\text{green}}^{2}}$$

In the laboratory, fish were then measured and dissected in order to collect standard length, total weight, and gonad weight. Each fish was eviscerated to estimate the somatic allotment, and gonadosomatic index (GSI) was calculated (respect to eviscerated weight) to estimate the reproductive allotment. To check whether the pigmentation of male caudal fin is a carotenoid‐based coloration, caudal fin pigments from ten randomly chosen fish samples were extracted in hexane to perform a spectrophotometric reading of the solution. The UV‐Visible spectra (collected at speed of scanning of 240 nm/min with the software UVW in Lab) were recorded in the range between 350 and 520 nm at room temperature, with the double beam scanning Perkin Elmer Lambda 35 UV/VIS spectrometer and then compared with the absorption spectra of four pigments: two carotenoids (betacarotene and lutein) and two pterins (drosopterin and xanthopterin), obtained from the literature (Johnson & Fuller, 2014; Zang, Sommerburg, & Van Kuijk, 1997).

### 2.1 Statistical analysis

A preliminary inspection of the data highlighted significant differences in standard length across habitat types (ANOVA: *F*_2,272_ = 33.97, *p* < .001). As the study of the variation in redness with size was not the object of this work, and to avoid the effect of size on the two biological predictors, data were log‐transformed and the linear least square regressions of standard length against redness, eviscerated weight, and GSI were performed. A significant relationship of redness (*F*_1,273_ = 48.92; *p* < .001) and eviscerated weight (*F*_1,273_ = 3084; *p* < .001) with standard length was found. Therefore, the following statistical analyses were performed on the residuals from these two regressions. Analysis of variance was used to test for differences in the mean values of size, redness, somatic investment, and GSI among habitats, after checking for normality and homogeneity of variance. Due to the low number of observations, differences in water turbidity and sediment chlorophyll concentration among habitats were tested using a Kruskal–Wallis test followed by Wilcoxon post hoc test. Pearson correlation was used to explore the relationship between redness of caudal fin and the two environmental variables, water turbidity, and chlorophyll concentration within the sediment.

A mixed‐effect model approach was adopted (Pinheiro & Bates, 2000; Pinheiro, Bates, DebRoy, & Sarkar, 2017) to deal with the unbalanced design and with the different potential source of variability in redness and to avoid multiple individual correlations. Following the protocol proposed by Zuur, Ieno, Walker, Saveliev, and Smith (2009), alternative variance structures and random terms were considered, comparing saturated models. The chosen random part of the model was the one containing the site as random term and an exponential function of the variance covariate (chlorophyll).

Once the “optimal” random structure was chosen, 13 (Table 1) different fixed part model formulations were compared using AIC. Each model contained different combinations of habitat characteristics, biological traits, or both. Wald test was used to assess the significance level of the terms included in the chosen model. This approach will allow to point out the significant effects of habitat type, water turbidity, sediment chlorophyll, and somatic/reproductive investment in explaining the variations in redness observed.

**Table 1:** List of the models fitted with the corresponding AIC values. The selected model is highlighted in bold (T—water turbidity, Chl—chlorophyll concentration in the sediment, W—eviscerated weight, G—gonadosomatic index, H—habitat, *S—*site)

Label	Model structure	*df*	AIC
m0	redness ~ H + *S*	6	325.93
m1	redness ~ H + T + Chl + *S*	8	306.89
m1.1	redness ~ H + T:H + Chl:H + *S*	12	302.45
m2	redness ~ H + W + G + *S*	8	328.70
m2.1	redness ~ H + W:H + G:H + *S*	12	332.34
m3	redness ~ H + T + Chl + W + G + *S*	10	308.07
m3.1	redness ~ H + T:H + Chl:H + W:H + G:H + *S*	18	309.23
m4	redness ~ H + T:H + Chl:H + W + G + *S*	14	304.33
m4.1	redness ~ H + T:H + Chl + W + G + *S*	12	310.81
m4.2	redness ~ H + T + Chl:H + W + G + *S*	12	304.41
m5	redness ~ H + T + Chl + W:H + G:H + *S*	14	305.73
**m5.1**	**redness ~ H + T + Chl + W:H + G +** ***S***	**12**	**302.14**
m5.2	redness ~ H + T + Chl + W + G:H + *S*	12	311.76

## 3 RESULTS

**Figure 2:**
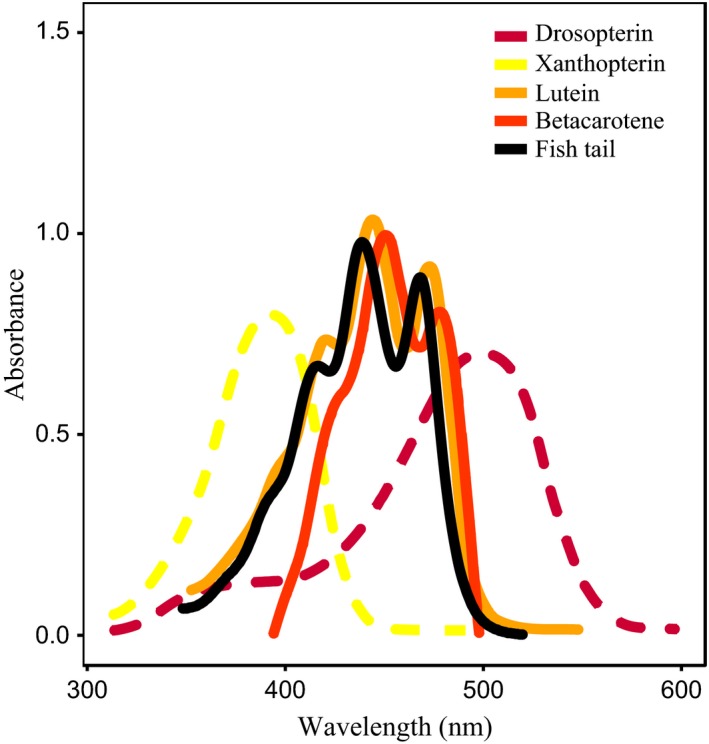
Absorption spectra of caudal fin extract in hexane compared with the spectra of two carotenoids (solid lines: lutein and β‐carotene from Zang et al., 1997) and two pterins (dashed lines: drosopterin and zanthopterin from Johnson & Fuller, 2014)

The spectrophotometric analysis of pigments extracted from caudal fin in 10 males of *A. fasciatus* confirmed the presence of carotenoids, showing the typical absorption spectrum (Figure 2) with two main peaks around 430–440 nm (mean absorption ± *SE* = 0.15 ± 0.03) and 460–470 nm (mean absorption ± *SE* = 0.13 ± 0.03).

**Figure 3:**
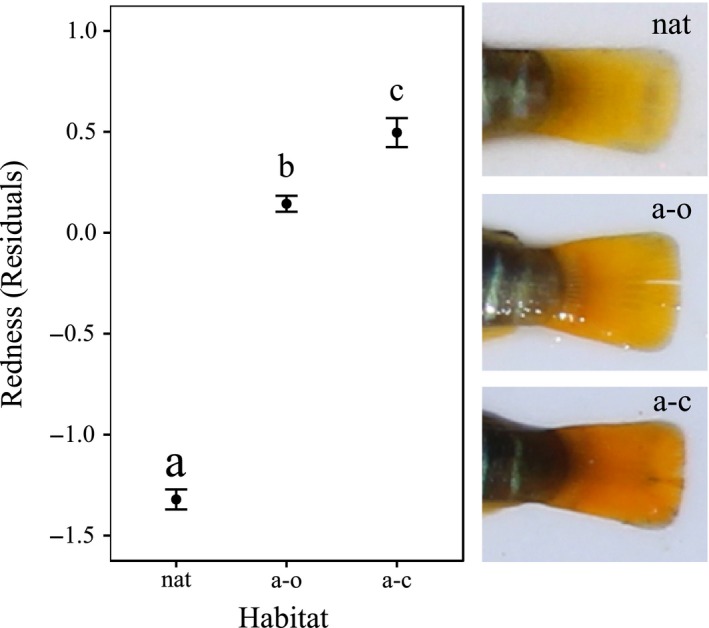
Mean residual values (±*SE*) of the intensity of caudal fins coloration (size‐corrected redness index) for male *A. fasciatus* in the three habitat types: natural salt marsh creeks (nat), artificial open creeks (a–o), and artificial closed creeks (a–c). Different letters above the habitat type indicate a significant difference at *p* < .05 according to the Tukey's HSD post hoc test

After correction for size effect (Figure 3), significant differences in caudal fin intensity of coloration were found among all the three habitat types (*F*_2,272_ = 193.8, *p* < .001; ANOVA followed by Tukey's HSD post hoc). The redness index showed the lowest values in the natural creeks, where a yellow coloration was observed. Conversely, the fins in artificial habitats showed higher values of the index, with fish expressing a more orange coloration in the closed systems than in the open ones.

**Figure 4:**
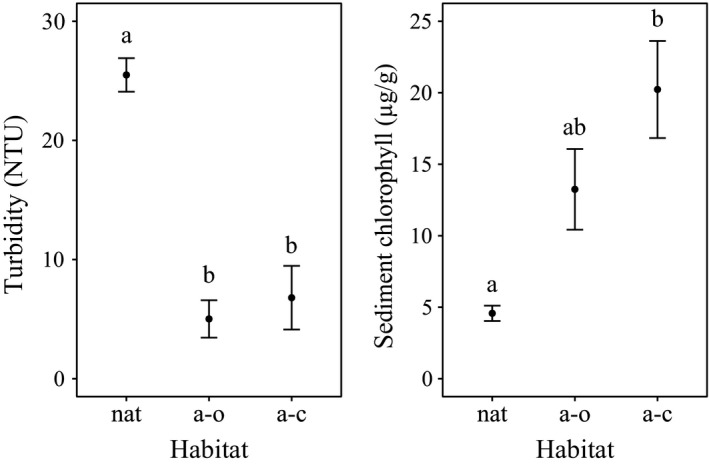
Mean values (± *SE*) of water turbidity (left) and sediment chlorophyll concentration (right) in the three habitat types: two natural salt marsh creeks (nat), four artificial open creeks (a–o), and four artificial closed creeks (a–c). Different letters above the habitat type indicate a significant difference at *p* < .05 according to the Wilcoxon post hoc test

The three habitat types investigated in this study showed different levels of water transparency and availability of carotenoid pigments. Natural creeks within salt marsh systems were characterized by a significantly higher turbidity respect to the two types of artificial habitats (*H*_2_ = 9.15, *p* < 05; Kruskal–Wallis followed by Wilcoxon post hoc, Figure 4). Considering the microphytobenthos standing crop, in the natural creeks, a significantly lower concentration of benthic chlorophyll was found than in the closed artificial systems (*H*_2_ = 9.91, *p* < .05; Kruskal–Wallis followed by Wilcoxon post hoc, Figure 4), while open systems showed intermediate values.

**Figure 5:**
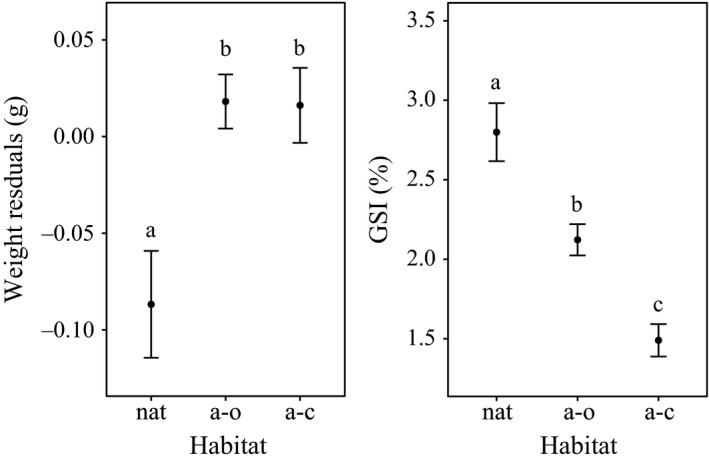
Mean residual values (± *SE*) of the somatic (left) and reproductive (right) allotment for male *A. fasciatus* in the three habitat types: natural salt marsh creeks (nat), artificial open creeks (a–o), and artificial closed creeks (a–c) according to the Wilcoxon post hoc test

Site‐averaged male caudal fin coloration, expressed by the redness index, showed a significant negative correlation (*r* = −.77, *t*_8_ = −3.44, *p* < .05; Pearson correlation) with water turbidity measured in the ten sites, and a significant positive correlation (*r* = .87, *t*_8_ = 4.90, *p* < .001; Pearson correlation) with the sediment chlorophyll. In the case of turbidity, observations were not well aligned as in the latter case, suggesting the presence of other uncontrolled factors influencing this relationship. No significant correlation was found between water turbidity and sediment chlorophyll concentration (*r* = −.46, *t*_8_ = −1.47, *p* = .18; Pearson correlation). Also, the somatic and reproductive allotment of *A. fasciatus* males differed across habitat types (Figure 5). The size‐corrected mean eviscerated weight was lower in natural creeks respect to the artificial habitats (*F*_2,272_ = 7.99, *p* < .001; ANOVA followed by Tukey's HSD post hoc), with no significant differences between open and closed systems. Reproductive allotment showed a different pattern, with significant differences in the gonadosomatic index across the three habitat types (*F*_2,272_ = 11.61, *p* < .001; ANOVA followed by Tukey's HSD post hoc).

Among the 13 models fitted, the lowest AIC values were for m.1 (AIC = 302.45) and m5.1 (AIC = 302.14) (Table 1). As the ΔAIC is too small, there is not enough support to identify a single best model on the basis of a statistical criterion. Considering the purposes of this work, the latter was chosen, not for the slightly lower AIC values, but mainly because it included in the fixed part both environmental and biological predictors.

**Figure 6:**
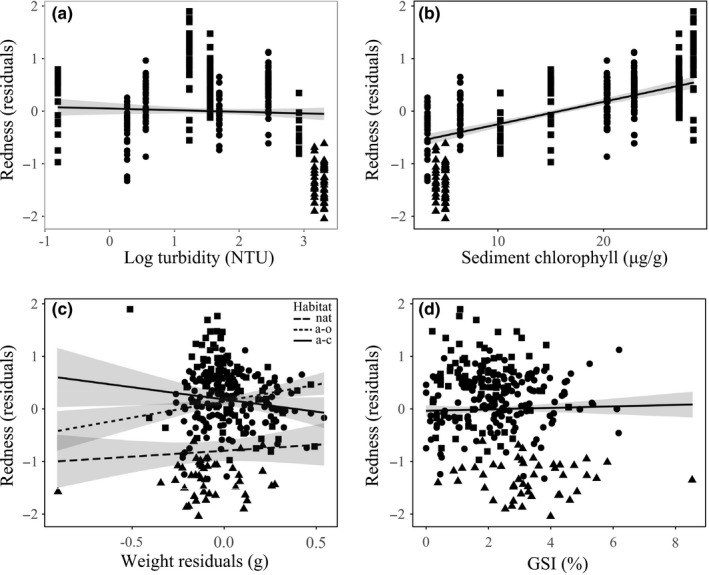
Relationships of the intensity of coloration (redness index) in male *A. fasciatus* caudal fins with: the two environmental variables, water turbidity (a) and sediment chlorophyll concentration (b), and with the two biological traits, somatic (c) and reproductive (d) allotments in the three habitat types: natural salt marsh creeks (triangles), artificial open creeks (circles), and artificial closed creeks (squares). The lines represent the prediction of the selected model (m5.1)

The selected model showed a significant effect of habitat type, water turbidity, chlorophyll concentration, and somatic weight on caudal fin coloration, while the GSI did not affect significantly redness (Table 2). In particular, redness values showed a slightly negative relationship with water turbidity (Figure 6a) and a positive relationship with sediment chlorophyll (Figure 6b). For the somatic weight, the best model structure included the interaction with the habitat type. Indeed, while a positive relationship between redness and eviscerated weight was found in natural and artificial open habitats, in the closed artificial creeks, this relationship turned out to be negative; in this habitat, fish with a more intense orange coloration showed a lower somatic investment respect to fish with a less colored caudal fin.

**Table 2:** Results of the Wald test for the selected model (m5.1)

	*df*	*F*	*p*
Intercept	1, 261	10.10	.002
Habitat	2, 5	207.27	<.001
Turbidity	1, 5	7.93	.037
Chlorophyll	1, 5	90.47	<.001
GSI	1, 261	0.29	.593
Weight : habitat	3, 261	3.37	.019

## 4 DISCUSSION

Results of this work suggested that the coloration of caudal fin in males *A. fasciatus* may be related to habitat characteristics and sediment chlorophyll concentration. Furthermore, the negative relationship between caudal fin redness and somatic weight observed in the artificial closed creeks suggested the possible presence of a trade‐off between these two variables.

In shallow water habitats such as the creeks considered in the present study, the microphytobenthic community plays a key role among primary producers (MacIntyre et al., 1996; Webster et al., 2002), may be representing the dominant source of carotenoids for the trophic chain. This could explain the strong significant correlation found between the redness of caudal fin and the benthic concentration of chlorophyll. Grether et al. (1999) found a strong relationship between carotenoid availability and the concentration of chlorophyll in the periphyton from the streams of Trinidad. Through the analysis of gut and skin pigments, these authors suggested how the carotenoid availability in the environment influenced the pigment deposition in the orange spot of male guppies.

To make effective such signal, it must be conveyed through the medium, that is water, in order to reach the receiver. In the aquatic environment, the effectiveness of visual signals can be deeply altered or weakened by water turbidity (Candolin et al., 2007; Endler, 1992; Fuller, 2002; Maan et al., 2010; Pauers, 2011). In the present study, water turbidity showed significant differences between natural and artificial habitats, and model predictions showed a significant effect of turbidity on caudal fin coloration. Yellow caudal fins were found in the turbid waters of natural creeks, while orange caudal fins in the artificial habitats, characterized by a higher water transparency. A selection for red pigmentations in transparent waters was observed, for example, in the threespine stickleback, *Gasterosteus aculeatus* (Reimchen, 1989), in the bluefin killifish *Lucania goodei* (Fuller & Travis, 2004), and in the African cichlid *Pseudocrenilabrus multicolour victoriae* (McNeil et al., 2016). Water turbidity could select for a yellow or an orange‐reddish coloration to maximize the visibility of the signal by a conspecific fish but not by potential predators; further studies are needed to assess how the yellow‐orange caudal fin of *A. fasciatus* is perceived by a conspecific observer or by a potential predator, taking into account also water optical properties.

Precedent studies focused on female *A. fasciatus*, from natural and artificial habitats of the Venice lagoon, showed how differences in mortality rates, as consequences of different habitat structures, shaped the life history strategy of the species, modulating the patterns of energy allocation (Brigolin et al., 2016). The trade‐off between somatic and reproductive compartments observed for female *A. fasciatus* seems to be present also in males. The reproductive investment was higher in the open systems (natural habitats) than in the closed systems (artificial closed habitats), while an opposite pattern was shown for the somatic investment. Artificial open systems showed an intermediate situation, somewhere in between the gradient of decreasing mortality from natural open creeks and closed artificial systems observed in precedent studies (Cavraro et al., 2014).

Secondary sexual traits can be considered a third body system other than the somatic and reproductive compartments. Even if carotenoid‐based coloration should represent an honest signal of an individual's health, according to the handicap principle, the sequestration of carotenoids into sexual ornamentations should be considered as a cost for an organism. This cost would lead to the emergence of a trade‐off with other physiological processes, such as antioxidant functions, hormone regulation, and immune defense (Clotfelter, Ardia, & McGraw, 2007; Olson & Owens, 1998). In particular, the immune system competes with ornaments for the available pool of immunostimulating carotenoids (Alonso‐Alvarez, Prez‐Rodriguez, Mateo, Chastel, & Vinuela, 2008; Vinkler & Albrecht, 2010). Through the mediation of testosterone, carotenoids would be used alternately in the pigmentation of secondary sexual characters or in self‐maintenance processes, with an energy expenditure detrimental on growth or other essential functions (Vinkler & Albrecht, 2010). For example, on a 10‐week period, male sticklebacks *Gasterosteus aculeatus*, raised by Frischknecht (1993), showed an inverse relationship between body growth and carotenoid‐based throat coloration. In the present study, a possible cost of using carotenoid pigmentation as a secondary sexual character emerged in the artificial closed systems, where, despite a higher availability of carotenoid sources and a higher redness, a negative relationship was observed between caudal fin redness and somatic allotment. Only within this habitat, the most intense coloration was shown by males with a low somatic investment, while heavier fish showed average values of redness comparable to those found in the other two habitat types. Carotenoid pigmentation should be an indicator of health (Olson & Owens, 1998). Indeed, in our results, a negative relationship between redness and somatic investment was found only in the closed artificial creeks, where the highest values of redness were found. Although not measured in the present work, precedent studies found higher densities in an artificial closed habitat, with a detrimental effect on growth rates (Cavraro, et al., 2014; Cavraro, Torricelli, et al., 2013). Indeed, high densities in a delimited space could determine competition for resources or favor the outbreak of diseases and parasite infections. Therefore, in this situation, carotenoid‐based pigmentation could represent a dishonest signal. No significant effect of GSI on redness was found in this study, probably because in *A. fasciatus* males, the gonadosomatic index was relatively low (2%–3% on average). Nevertheless, in this species, reproductive costs should not be attributable only to the development of gonads or secondary sexual characters. Indeed, males of *A. fasciatus* exhibit an intense and elaborated courtship behavior (Cavraro, Torricelli, et al., 2013; Malavasi et al., 2010). Therefore, also this frenetic activity may determine a relevant energy expenditure that could influence the overall allocation of resources, particularly in the artificial closed habitats, where fish would be free to display their sexual character in a low‐predation environment. Further manipulative studies would be necessary to provide useful information about this topic.

## 5 CONCLUSIONS

This study investigated, for the first time as concerns a transitional water ecosystem, how habitat characteristics can influence the expression of a carotenoid‐based secondary sexual trait, such as the caudal fin of males *A. fasciatus*. Differences in coloration intensity observed were mainly related to the availability of carotenoids in the environment. Furthermore, results suggested the existence of a trade‐off between the use of carotenoids as pigments and the somatic investment. The analytical identification and quantification of the pigments, both in the environment and in the fish tissue, would help to further analyze this topic, providing useful information about the physiological and ecological roles of carotenoids in fish.

## CONFLICT OF INTEREST

None declared.

## AUTHORS’ CONTRIBUTIONS

FC and SM conceived the ideas and designed methodology; FC collected the data; FC and MZ analyzed the data; FC and SM led the writing of the manuscript. All authors contributed critically to the drafts and gave final approval for publication.
