# Supplementary figures and images for: Habitat constraints on carotenoid‐based coloration in a small euryhaline teleost
Source: Ecol Evol. 2018 Apr 2;8(9):4422–30. doi: 10.1002/ece3.4003 (PMC5938449; doi:10.1002/ece3.4003)

Appendix


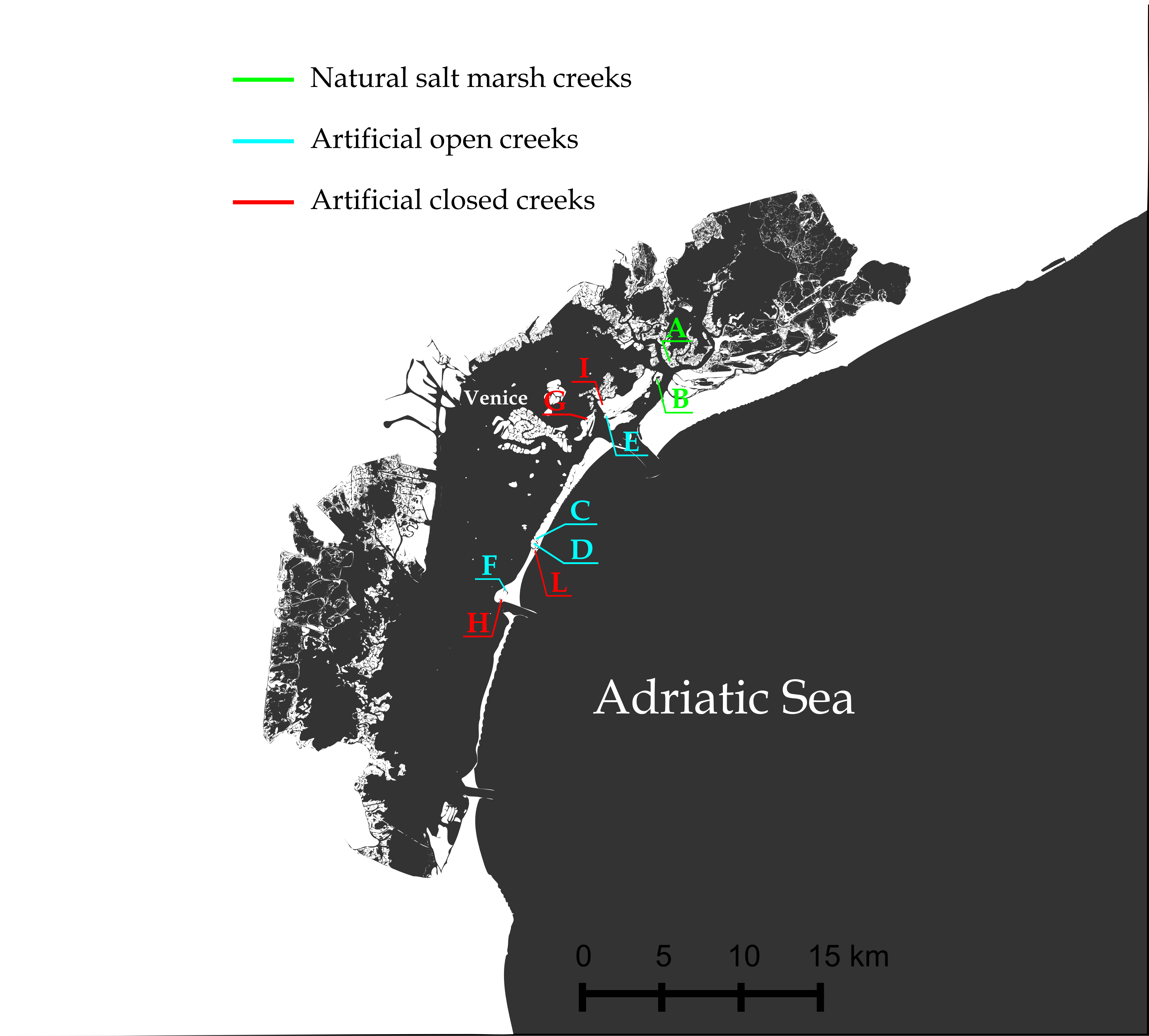

Supplement: Supplementary file 1 [file ECE3-8-4422-s001.docx]
